# Prediction and Prevention of Acute Kidney Injury after Cardiac Surgery

**DOI:** 10.1155/2016/2985148

**Published:** 2016-06-23

**Authors:** Su Rin Shin, Won Ho Kim, Dong Joon Kim, Il-Woo Shin, Ju-Tae Sohn

**Affiliations:** ^1^Department of Internal Medicine, Samsung Changwon Hospital, Sungkyunkwan University School of Medicine, Changwon 51353, Republic of Korea; ^2^Department of Internal Medicine, College of Medicine, Kangwon National University, Chuncheon 24341, Republic of Korea; ^3^Department of Anesthesiology and Pain Medicine, Seoul National University Hospital, Seoul 03080, Republic of Korea; ^4^Department of Anesthesiology and Pain Medicine, Samsung Changwon Hospital, Sungkyunkwan University School of Medicine, Changwon 51353, Republic of Korea; ^5^Department of Anesthesiology and Pain Medicine, Gyeongsang National University Hospital, Gyeongsang National University School of Medicine, Jinju 52727, Republic of Korea

## Abstract

The incidence of acute kidney injury after cardiac surgery (CS-AKI) ranges from 33% to 94% and is associated with a high incidence of morbidity and mortality. The etiology is suggested to be multifactorial and related to almost all aspects of perioperative management. Numerous studies have reported the risk factors and risk scores and novel biomarkers of AKI have been investigated to facilitate the subclinical diagnosis of AKI. Based on the known independent risk factors, many preventive interventions to reduce the risk of CS-AKI have been tested. However, any single preventive intervention did not show a definite and persistent benefit to reduce the incidence of CS-AKI. Goal-directed therapy has been considered to be a preventive strategy with a substantial level of efficacy. Many pharmacologic agents were tested for any benefit to treat or prevent CS-AKI but the results were conflicting and evidences are still lacking. The present review will summarize the current updated evidences about the risk factors and preventive strategies for CS-AKI.

## 1. Introduction

Postoperative acute kidney injury (AKI) is now well recognized as a frequent and serious postoperative complication after cardiac surgery. It is one of the most significant causes of morbidity and mortality in patients undergoing cardiac surgery. AKI could even evolve to chronic kidney disease [1, 2]. According to the definition and surgery type, the incidence of postoperative AKI varies widely, from 33% to 94% [2–5]. Even small transient increases in serum creatinine that occur in the postoperative period are associated with a decreased patient survival [6]. As there is no effective therapy available for AKI after cardiac surgery [7, 8], there is an increasing desire to decrease postoperative AKI to improve clinical outcomes in patients undergoing cardiovascular surgery.

## 2. Definition and Classification

The Acute Dialysis Quality Initiative (ADQI) work group introduced a multilevel classification system for AKI, RIFLE classification in 2004 (Table 1) [9]. This investigators group subsequently published AKIN (acute kidney injury network) criteria which discarded chronic criteria and estimated glomerular filtration rate (GFR) [10]. The same study group recently suggested KDIGO (kidney disease: improving global outcomes) criteria which added RRT and 12 hours of anuria as criteria for Stage 3 AKI. The RIFLE and AKIN criteria are well established criteria [11, 12] but KDIGO criteria needed to be further validated to replace the previous two criteria [13].

## 3. Etiology and Risk Prediction of CS-AKI

The etiology of cardiac surgery-associated AKI (CS-AKI) after cardiac surgery is multifactorial, including ischemia-reperfusion injury, inflammation, and oxidative stress [14]. Hemodynamic parameter should also be considered for prerenal AKI, because kidney is susceptible to ischemic damage during renal hypoperfusion by perioperative low cardiac output syndrome and possible cardiogenic shock [15]. As no efficacious therapy for AKI is available at present [7, 8], identifying risk factors and a reduction of the risk of AKI would be beneficial [16, 17]. Several specific risk scores have been developed for AKI or postoperative dialysis following cardiac surgery, and these scores have demonstrated good performance in their study populations (Table 2) [16, 18–23]. The risk factors of these risk scores vary from study to study with some overlap. Some of reported risk factors including preoperative anemia, red blood cells transfusions, and preoperative hypoalbuminemia were considered to be potentially modifiable [17, 24], but prospective clinical trials with enough power are required to test whether modification of the modifiable risk factors could really reduce the incidence of AKI. Serum albumin may have a renoprotective effect by improving renal perfusion, inhibiting apoptosis of renal tubular cells, and promoting the proliferation of renal tubular cells [25–27]. However, a recent propensity score matched retrospective study has reported that albumin administration was associated with a dose-dependent risk of AKI [28]. Since Song et al. [29] first reported that prophylactic administration of erythropoietin in patients scheduled for elective CABG can prevent AKI, several randomized controlled trials (RCTs) have been performed in adult patients undergoing cardiac surgery. However, the results were controversial [30].

Cardiopulmonary bypass (CPB) itself has been regarded as a cause of developing CS-AKI. Blood cells are exposed to nonphysiologic surfaces of CPB circuit and shear forces, resulting in cell lysis and systemic and renal interstitial inflammation [31]. This inflammation causes the systemic inflammatory response syndrome (SIRS), which has been considered as one of the important pathologic mechanisms of CS-AKI. Changes of physiologic pulsatile to nonphysiologic linear flow during CPB exacerbate organ injury presumably by elevating peripheral vascular resistance, leading to poor microcirculation, and increasing tissue edema [31]. A recent retrospective study reported the perioperative neutrophil-lymphocyte ratio as a predictor of CS-AKI, highlighting the role of SIRS in the pathogenesis of CS-AKI [32]. As a consequence, a long operation time and cardiopulmonary bypass (CPB) duration were identified as a risk factor for AKI [19, 33–38]. Hypoxic renal injury during deep hypothermic circulatory arrest (DHCA) time has been thought to be a risk factor for AKI [39, 40], so a long DHCA time may result in a higher incidence of AKI. However, operation time including CPB and DHCA time is not considered to be modifiable.

A previous RCT demonstrated that general anesthesia with propofol was associated with significant reduction in the incidence of CS-AKI compared with sevoflurane anesthesia [41]. However, due to small sample size and single-center design, large multicenter trial is required to confirm this result. There have been controversies over whether perioperative aprotinin use is associated with renal dysfunction [42–44]. Aprotinin was withdrawn from the market since the BART study [44], but another study suggested that the administration of aprotinin does not increase the risk of renal dysfunction [45].

## 4. Biomarkers of CS-AKI

Biomarkers of renal injury have been extensively studied recently because these markers can provide early detection of AKI and prognostic value. Most frequently studied promising biomarkers are neutrophil gelatinase-associated lipocalin (NGAL) and interleukin-18 (IL-18) [46, 47]. Serum cystatin C is also suggested to be a useful predictor of CS-AKI [48, 49].

## 5. Preventive Interventions by Risk Factor Modification and Hemodynamic Optimization

There are plenty of studies reporting preventive interventions to mitigate CS-AKI. Numerous small RCTs corroborated the preventive effect of risk factor modifications, but their effects were mostly inconsistent and level of evidence was low. Hemodynamic optimization showed relatively consistent promising results [50]. Prevention of AKI appears to be best delivered by a multimodal approach, because renal injury can occur from multifactorial etiologies.

### 5.1. Hemodynamic Optimization or Goal-Directed Therapy

Recent retrospective cohort study reported that AKI is associated with intraoperative period of mean arterial pressure (MAP) less than 55 and less than 60 mmHg [51]. This result requires a clinical trial to determine whether interventions that prevent intraoperative hypotension and maintain MAP strictly above 55 and 60 mmHg could help reduce CS-AKI.

Renal perfusion may be preserved by maintaining adequate intravascular volume and cardiac output, mainstay of the protocolized patient care, so-called “goal-directed therapy.” These strategies also called “hemodynamic optimization” refer to the perioperative monitoring and maintaining specific hemodynamic goals by means of fluid, transfusion, and inotropes. A previous meta-analysis of 4,220 surgical patients tested whether hemodynamic optimization could reduce the incidence of postoperative AKI and concluded that surgical patients receiving perioperative hemodynamic optimization are at decreased risk of renal dysfunction [50]. However, there have been concerns that the administration of hydroxyethyl starch as part of goal-directed therapy is associated with AKI [52–54].

### 5.2. Fluid, Colloid Administration, and Transfusion

Chloride-rich fluids are associated with worse clinical outcomes compared with balanced solutions [55–57]. Analysis of more than 30,000 adult patients undergoing major abdominal surgery showed that patients receiving 0.9% saline were prone to require RRT and had greater transfusion requirements and more infectious complications compared to those receiving balanced solution [56]. Another prospective study reported by Yunos et al. with more than 1,500 patients in ICU reported that patients receiving chloride restricted solutions had less AKI and required RRT less often than those receiving chloride-rich solutions [57]. The effect of fluid balance on CS-AKI is studied. In a recent single-center retrospective analysis, postoperative fluid overload was associated with poor short-term outcome of patients with CS-AKI [58].

Multicenter RCTs were published to test whether use of hydroxyethyl starch (colloids) compared with crystalloid solutions alters mortality and renal function in ICU patients with or without sepsis [59, 60]. They found no difference in short-term mortality and more patients receiving colloids were treated with renal replacement therapy. Recent meta-analyses confirmed that clinical use of colloids was associated with increased risk of AKI and dialysis in critically ill patients [52–54]. Perioperative transfusion is associated with high risk of CS-AKI, but a direct causal relationship between red blood cell transfusion and AKI has not been confirmed [61].

### 5.3. CPB Parameters

Hypotension during CPB was not suggested to be associated with development of CS-AKI [62]. However, a prospective observational study showed that mean arterial pressure below the cerebral autoregulation threshold during CPB is independently associated with CS-AKI [63]. Hemodilution during CPB is considered to contribute to CS-AKI by impairment of oxygen delivery to the hypoxic kidney medulla. A retrospective study observed that intraoperative hematocrit <24% was associated with increased risk of CS-AKI [64], which was also reported in a recent prospective study [65]. The technology of pulsatile CPB was developed to mimic physiologic blood flow. However, the theoretical benefit of pulsatile CPB to improve microcirculation has not yet been proved in a randomized observational study [31], while it showed some benefit in terms of better maintenance of glomerular filtration rate and lower renal tissue injury measured by kidney injury markers including NGAL [66]. Intra-aortic balloon pump (IABP) can produce pulsatile perfusion during CPB in high-risk patients and a previous RCT enrolling 501 coronary artery bypass graft (CABG) surgery patients demonstrated that IABP-induced pulsatile CPB improved whole-body perfusion and reduced endothelial activation [67]. However, a recent animal study raised concern regarding using IABP to achieve pulsatile perfusion by showing the lowered aortic pressure in the distal aorta and the impaired renal tissue perfusion [68].

### 5.4. Metabolic Control

Tight blood glucose control (blood glucose <110 mg/dL) was associated with a reduction in the incidence of postoperative mortality and hemodialysis in cardiac surgical patients [69, 70]. However, these positive findings were questioned by a recent multicenter trial and meta-analysis in critically ill patients [71, 72]. A meta-analysis pointed out that patients in surgical ICUs appear to benefit from intensive insulin therapy [72]. Hyperuricemia has been associated with an increased risk of CS-AKI [73]. However, a small RCT failed to demonstrate the effect of uric acid lowering therapy on the prevention of CS-AKI [74]. More studies are required to clarify exact relationship between hyperuricemia and CS-AKI.

### 5.5. Avoid Nephrotoxic Agents

Preoperative combined administration of angiotensin-converting enzyme (ACE) inhibitors, angiotensin receptor blockers (ARB), diuretics, or nonsteroidal anti-inflammatory drugs is associated with increased risk of AKI [75, 76].

### 5.6. Early Initiation of Renal Replacement Therapy

Elahi et al. suggested that early continuous renal replacement therapy (CRRT) may improve mortality and mortality in severe CS-AKI [77]. A meta-analysis from 15 studies and 2955 patients reported that early CRRT could reduce the mortality of patients with AKI [78].

## 6. Pharmacologic Renal Protection

Although a variety of studies have been published, there is no consistent evidence with sufficient power to support routine use of any specific pharmacological drug in preventing CS-AKI (Table 3).

### 6.1. Dopamine and Fenoldopam

Dopamine and fenoldopam, a selective dopamine-1 receptor agonist, are expected to protect renal function due to its renal vasodilatory and natriuretic effect. However, dopamine does not seem to prevent or ameliorate CS-AKI [79]. A previous RCT enrolling 80 patients undergoing cardiac surgery has shown that fenoldopam prevented AKI and major morbidity in the subgroup of patients requiring inotropic support [80]. A meta-analysis from 6 RCTs showed that fenoldopam significantly reduced the incidence of AKI, but no effect on RRT, survival, and length of ICU/hospital stay [81]. Large, multicenter, adequately powered RCTs are required to confirm favorable effect to reduce the risk of AKI.

### 6.2. Statins

In addition to lipid-lowering properties, statin (3-hydroxy 3-methylglutaryl-CoA reductase inhibitors) possesses antioxidant properties and improves endothelial function and anti-inflammatory action, whose properties provide potential renoprotective effects. A retrospective analysis suggested that early postoperative statin therapy is associated with a lower incidence of CS-AKI [82]. However, a large retrospective study and a multicenter prospective cohort study failed to prove that preoperative statin use can decrease the incidence of CS-AKI [83, 84]. Meanwhile, continuing statin before surgery was associated with a lower risk of elevation of AKI biomarkers [84]. Large RCTs with adequate sample size are still required.

### 6.3. Dexmedetomidine

Dexmedetomidine is a highly selective alpha-2 agonist and has been shown to protect renal function in animal studies by stabilizing sympathetic activation and anti-inflammatory effects and attenuating ischemia/reperfusion injury. A retrospective analysis of 1,133 patients reported that post-bypass dexmedetomidine use was associated with a reduction in incidence of CS-AKI [85]. A recent triple-blinded RCT found that dexmedetomidine infusion for sedation after CABG under CPB can decrease blood NGAL levels for the first postoperative day in a dose-dependent manner [86]. Cho et al. [87] conducted a placebo-controlled RCT in 200 patients undergoing valvular heart disease and found that dexmedetomidine infusion reduced both incidence and severity of AKI. Current evidences of dexmedetomidine are promising, but more RCTs are still required to test the effect of dexmedetomidine on the incidence of CS-AKI.

### 6.4. Sodium Bicarbonate

Although a small RCT suggested that urinary alkalinization may protect against CS-AKI [88], following large trial and meta-analysis concluded that sodium bicarbonate infusion was not associated with lower incidence of AKI [89, 90]. Recent meta-analyses concluded that urinary alkalinization may reduce severe AKI in elective CABG [89, 91].

### 6.5. Mannitol

Mannitol, an osmotic diuretic, has been evaluated for its renoprotective property. The effect of mannitol used in the priming fluid for CPB on renal function was evaluated in previous RCTs, but no difference was observed [92]. A recent prospective observational study has found that mannitol induces a renal vasodilation and increased renal blood flow with no change in filtration fraction or the renal oxygen supply/demand relation in patients with postoperative AKI [93]. More studies are required regarding the therapeutic role of mannitol.

### 6.6. N-Acetylcysteine

N-acetylcysteine is an antioxidant and free radical scavenger. It has been studied for its potential role to reduce renal oxidative injury that contributes to CS-AKI. However, a recent meta-analysis showed that evidences do not support routine use of N-acetylcysteine to reduce CS-AKI [94].

### 6.7. Atrial Natriuretic Peptide (ANP) and Brain Natriuretic Peptide (BNP)

ANP and BNP block the renin-angiotensin-aldosterone system and induce renal arterial vasodilation. Previous studies have shown prophylactic use of ANP or BNP during cardiac surgery increased GFR and urine output and decreased AKI incidence [95, 96]. A meta-analysis reported that BNP decreased AKI incidence by 10% [97]. In a RCT of nesiritide, the recombinant human BNP, nesiritide reduced the incidence of AKI compared to controls [98]. However, well-designed, large multicenter RCTs are still required to assess the effect of ANP and BNP on preventing and treating CS-AKI.

### 6.8. Anti-Inflammatory Strategies

To modulate inflammatory response that contribute to the pathogenesis of AKI, anti-inflammatory strategies including glucocorticoid administration, miniaturized extracorporeal circuit, and the use of leukocyte filter have been investigated. A meta-analysis reported that, among these three interventions, only leukocyte filter application reduced renal injury in cardiac surgery patients [99]. However, the authors claimed the necessity of large well-powered trials with uniform and accepted AKI definition.

## 7. Conclusions

AKI is a frequent and severe complication after cardiac surgery that is associated with longer hospital stay and increased short- and long-term mortality. Although there are extensive studies to find interventions to prevent or reduce CS-AKI, preventive strategies are limited and the evidences are still lacking. Considering the multifactorial etiology of CS-AKI, it is less likely that any single intervention can reduce its incidence. Prevention of CS-AKI may require a multimodal approach such as protocolized therapy or combination of several preventive modalities. In addition, any multimodal approach should consider both patient and surgical parameters.

## Figures and Tables

**Table 1:** RIFLE, AKIN, and KDIGO classification for AKI diagnosis.

RIFLE		AKIN		KDIGO		Urine output^*∗*^
Criteria	Creatinine definition	Criteria	Creatinine definition	Criteria	Creatinine definition	
Risk	≥1.5-fold increase from baseline SCr or decrease in GFR ≥25%	Stage 1	≥0.3 mg/dL increase or ≥1.5-fold increase from baseline SCr within 48 hrs	Stage 1	≥0.3 mg/dL increase within 48 hrs or 1.5–1.9 times baseline within 7 days	<0.5 mL/kg/h for >6 hours

Injury	≥2-fold increase from baseline SCr or decrease in GFR ≥50%	Stage 2	≥2-fold increase from baseline SCr	Stage 2	2.0–2.9 times baseline within 7 days	<0.5 mL/kg/h for 12 hours

Failure	≥3-fold increase from baseline SCr or increase to ≥4 mg/dL or decrease in GFR ≥75%	Stage 3	≥3-fold increase from baseline SCr or increase to ≥4.0 mg/dL with an acute increase of >0.5 mg/dL or initiation of RRT	Stage 3	≥3 times baseline within 7 days or increase to ≥4.0 mg/dL with an acute increase of >0.5 mg/dL or initiation of RRT	<0.3 mL/kg/h for 24 hours or anuria for >12 hours

RIFLE: risk, injury, failure, loss, end-stage kidney disease; AKIN: acute kidney injury network; KDIGO: kidney disease: improving global outcomes; SCr: serum creatinine; GFR: glomerular filtration rate; RRT: renal replacement therapy.

^*∗*^Urine output criteria are common to three definitions.

**Table 2:** Risk scoring models for cardiac surgery-associated AKI.

Variables	Thakar et al. [16]	Mehta et al. [18]	Wijeysundera et al. [20]	Aronson et al. [21]	Palomba et al. [19]	Brown et al. [22]	Parolari et al. [23]
*Demographics*							
Age		0		0	0	0	0
Gender	0					0	

*Baseline medical conditions*							
Hypertension						0	
Pulse pressure hypertension				0			
Diabetes mellitus	0	0	0			0	0
Preoperative renal dysfunction	0	0	0	0	0		0
Congestive heart failure	0			0		0	
Decreased LVEF	0		0				
Advanced NYHA class		0			0		
Prior myocardial infarction		0		0			
Chronic lung disease	0	0					
Preoperative IABP	0		0			0	
Prior cardiac surgery	0	0	0			0	
Emergency operation	0		0				
Preoperative neutrophilia						0	

*Intraoperative variables*							
Increased CPB time				0	0		0
Two inotropes required				0			0
Transfusion							0
Diuretics use							0

*Postoperative variables*							
CVP > 14 cm H_2_O					0		
Low cardiac output					0		
Inotrope/vasoconstrictor use							0
Transfusion							0
Diuretics/antiarrhythmics use							0

NYHA: New York Heart Association; CPB: cardiopulmonary bypass; CVP: central venous pressure; IABP: intra-aortic balloon pump; LVEF: left ventricular ejection fraction.

**Table 3:** Meta-analyses regarding pharmacologic renal protection for preventing acute kidney injury after cardiac surgery.

Agent	Author/ publication year	Study type (RCT/ observational study)	Outcome	Patients (Drug/control, *n*)	Pooled effect size (OR, 95% CI)	*p* value	*I* ^2^ (heterogeneity)
Dopamine	Zacharias et al. 2013 [79]	10	AKI	541	1.36 (0.44, 4.23)	Not reported	Not reported
Fenoldopam	Zangrillo et al. 2012 [81]	5 (5/0)	AKI	202/207	0.41 (0.23, 0.74)	0.003	0%
Fenoldopam	Zangrillo et al. 2012 [81]	4 (4/0)	RRT	183/188	0.67 (0.10, 4.48)	0.68	62%
Statin	Liakopoulos et al. 2008 [100]	5 (2/3)	AKI	4236/2172	0.78 (0.46, 1.31)	0.34	58.3%
Sodium bicarbonate	Bailey et al. 2015 [89]	3 (3/0)	*∗*	877	1.11 (0.77, 1.60)	0.45	Not reported
Sodium bicarbonate	Tie et al. 2014 [91]	5 (5/0)	AKI	1079	0.99 (0.78, 1.24)	0.911	56.1%
Mannitol	Yang et al. 2014 [101]	4 (4/0)	†	94/93	−2.35 (−7.46, 2.75)	0.37	0%
N-acetylcysteine	Ho and Morgan 2009 [94]	10	RRT	485/487	1.04 (0.45, 2.37)	0.9	3.3%
N-acetylcysteine	Patel et al. 2011 [97]	4 (4/0)	AKI	453/450	0.86 (0.66, 1.13)	0.29	0%
N-acetylcysteine	Patel et al. 2011 [97]	7 (7/0)	RRT	503/499	0.98 (0.50, 1.92)	0.96	5%
Atrial natriuretic peptide	Patel et al. 2011 [97]	1 (1/0)	AKI	251/253	0.35 (0.23, 0.76)	0.005	One study
Atrial natriuretic peptide	Patel et al. 2011 [97]	5 (5/0)	RRT	427/426	0.24 (0.10, 0.56)	0.001	0%
Brain natriuretic peptide	Patel et al. 2011 [97]	2 (2/0)	AKI	186/187	0.40 (0.21, 0.76)	0.005	40%
Brain natriuretic peptide	Patel et al. 2011 [97]	2 (2/0)	RRT	64/69	0.80 (0.18, 3.64)	0.78	0%
Steroids adults	Scrascia et al. 2014 [99]	7 (7/0)	AKI	291/289	1.13 (0.53, 2.43)	Not reported	18.3%
MECC	Scrascia et al. 2014 [99]	6 (6/0)	AKI	391/427	0.47 (0.18, 1.25)	Not reported	0%
Leukofiltration	Scrascia et al. 2014 [99]	4 (4/0)	AKI	157/157	0.18 (0.05, 0.64)	Not reported	0%

AKI: acute kidney injury; RRT: renal replacement therapy.

^*∗*^Postoperative increase in serum creatinine concentration of greater than 25% or 0.5 mg/dL within the first five postoperative days.

^†^Change of serum creatinine concentration.
